# Efficacy and safety of bosentan-related therapy in neonates with persistent pulmonary hypertension of the newborn: a systematic review and meta-analysis

**DOI:** 10.3389/fped.2026.1836276

**Published:** 2026-06-03

**Authors:** Guanghong Li, Xiaoqun Du, Weibin Luo, Junhua Wei, Huiyi Huang

**Affiliations:** Department of Pediatrics, Huadu District People’s Hospital of Guangzhou, Guangzhou, China

**Keywords:** bosentan, mechanical ventilation, neonate, persistent pulmonary hypertension of the newborn, pulmonary artery pressure, safety

## Abstract

**Objective:**

To evaluate the efficacy and safety of bosentan-related therapy in neonates with persistent pulmonary hypertension of the newborn (PPHN).

**Methods:**

PubMed, Web of Science, the Cochrane Library, and ClinicalTrials.gov were searched from inception to March 2026 for clinical studies of bosentan in neonates with PPHN (CRD420261338730). Two reviewers independently screened studies, extracted data, and assessed risk of bias. Meta-analysis was performed using RevMan 5.4. Outcomes included treatment failure, change in pulmonary artery pressure, length of hospital stay, duration of mechanical ventilation, absolute tricuspid regurgitation values at 72 h, reduction in tricuspid regurgitation at 72 h, and safety.

**Results:**

Nine studies were included from 187 identified records. Bosentan-related therapy was associated with a lower treatment failure rate than control treatment (RR: 0.27, 95% CI: 0.14–0.51; *P* < 0.0001). In haemodynamic outcomes, bosentan-related therapy was associated with lower absolute tricuspid regurgitation values at 72 h (MD: −9.15, 95% CI: −12.79–−5.52; *P* < 0.00001) and greater reduction in tricuspid regurgitation at 72 h (MD: −6.42, 95% CI: −11.30–−1.54; *P* = 0.01). Reduction in pulmonary artery pressure also favoured bosentan-related therapy, although the difference was of borderline significance (MD: −2.69, 95% CI −5.40–0.01; *P* = 0.05). By contrast, bosentan-related therapy was not associated with a significant reduction in length of hospital stay (MD: −1.06, 95% CI −2.38–0.27; *P* = 0.12), and duration of mechanical ventilation was longer in the bosentan-related therapy group (MD: 2.04, 95% CI 1.26–2.82; *P* < 0.00001). Reported adverse events included hypotension, anaemia, oedema, vomiting, and hepatic abnormalities, including elevated liver enzymes or abnormal liver function. However, hepatic safety could not be quantitatively assessed because liver-related adverse events were inconsistently defined and reported across studies.

**Conclusion:**

Bosentan-related therapy may be associated with a lower risk of treatment failure and favorable changes in echocardiography-derived pulmonary pressure estimates in neonates with PPHN. However, current evidence does not support a clear benefit in harder clinical outcomes such as length of hospital stay, and no reduction in duration of mechanical ventilation was observed. Short-term safety appears acceptable, but caution remains warranted.

**Clinical Trial Registration:**

https://www.crd.york.ac.uk/prospero/display_record.php?ID=CRD420261338730. PROSPERO: CRD420261338730

## 1 Introduction

Persistent pulmonary hypertension of the newborn (PPHN) is a serious cardiopulmonary disorder characterized by persistently elevated pulmonary vascular resistance after birth, resulting in failure of the normal transition from fetal to postnatal circulation, right-to-left shunting at the atrial and/or ductal level, and severe hypoxemia (1). PPHN remains a major cause of morbidity and mortality in neonatal intensive care, with an estimated incidence of 1–2 per 1,000 live births, and continues to pose substantial challenges for acute management and long-term outcomes (2).

Current treatment for PPHN is based on management of the underlying cause and comprehensive supportive care, including optimization of oxygenation and ventilation, lung recruitment, administration of surfactant when indicated, maintenance of systemic hemodynamic stability, and correction of acidosis. On this basis, inhaled nitric oxide (iNO), as a selective pulmonary vasodilator, is considered a key first-line therapy for PPHN (3). However, important unmet clinical needs remain. Some infants show an inadequate or unsustained response to iNO, and a substantial proportion fail to achieve meaningful improvement in oxygenation. In severe or refractory cases, escalation to extracorporeal membrane oxygenation may be required, which is resource-intensive and not universally available. In addition, limited access to iNO and extracorporeal support in resource-constrained settings further restricts optimal management of PPHN (3, 4).

Accumulating evidence suggests that the endothelin pathway plays an important role in the pathogenesis of PPHN, with significantly increased plasma endothelin levels reported in affected neonates (5). Bosentan is an oral dual endothelin receptor antagonist that blocks both endothelin A and endothelin B receptors, thereby attenuating endothelin-mediated pulmonary vasoconstriction, promoting pulmonary vasodilation, and reducing pulmonary arterial pressure. The drug was originally approved by the US Food and Drug Administration in 2001 for the treatment of pulmonary arterial hypertension. Although bosentan has not been specifically approved for neonatal PPHN, its well-established pharmacologic rationale and prior use in pulmonary arterial hypertension have led to increasing off-label use in this setting, particularly when iNO is unavailable or insufficiently effective (4). Nevertheless, while bosentan may provide pulmonary vasodilatory benefit, it may also be associated with adverse effects such as systemic hypotension, elevated aminotransferase levels, and even hepatic injury. Its efficacy and safety in PPHN therefore remain incompletely defined (6). Accordingly, we performed a meta-analysis of the available clinical evidence to systematically evaluate the efficacy and safety of bosentan in the treatment of PPHN and to provide an evidence base for clinical decision-making and future research.

## 2 Materials and methods

### 2.1 Search strategy

This study was conducted in accordance with the Preferred Reporting Items for Systematic Reviews and Meta-Analyses (PRISMA) 2020 statement (7) and was registered in PROSPERO (CRD420261338730). A systematic literature search was performed in PubMed, Web of Science, the Cochrane Library, and ClinicalTrials.gov from database inception to March 2026. The search strategy combined controlled vocabulary terms and free-text terms and was constructed around the disease of interest, the study population, and the intervention. Chinese search terms included “bosentan”, “newborn”, and “persistent pulmonary hypertension”, whereas English search terms included bosentan, Ro47–0203, Tracleer, newborn, neonate, infant, persistent pulmonary hypertension of the newborn, PPHN, and persistent fetal circulation. The search strategy was adapted as appropriate for the specific requirements of each database. To minimize the risk of missing eligible studies, the reference lists of included articles and relevant reviews were also screened manually. The detailed search strategies for each database are provided in the Supplementary Table S1.

### 2.2 Eligibility criteria

The inclusion criteria were as follows:

① Study design: published randomized controlled trials (RCTs) in English;
② Participants: neonates diagnosed with persistent pulmonary hypertension of the newborn (PPHN) according to the relevant guidelines issued by the American Heart Association and the American Thoracic Society (8), For the purpose of this research, PPHN was operationally defined as a clinical syndrome of hypoxemic respiratory failure during the neonatal period, supported by echocardiographic findings suggestive of elevated pulmonary arterial pressure, such as tricuspid regurgitation-derived pulmonary pressure estimates, interventricular septal flattening, or right-to-left/bidirectional shunting across the ductus arteriosus or foramen ovale.
③ Interventions: patients in the intervention group received bosentan monotherapy or bosentan in combination with conventional therapy, whereas patients in the control group received placebo, conventional therapy, or other pharmacologic treatments not containing bosentan;
④ Outcomes: studies were required to report at least one prespecified outcome, including treatment failure rate, pulmonary artery pressure or echocardiography-derived pulmonary pressure estimates, tricuspid regurgitation-derived parameters, length of hospital stay, duration of mechanical ventilation, or adverse events. Oxygenation variables, including arterial partial pressure of oxygen and oxygen saturation, were considered for extraction when reported but were not included as mandatory eligibility outcomes or quantitatively synthesized because of inconsistent reporting across studies.

The exclusion criteria were as follows:

① Studies in which hypoxemia was primarily attributable to cyanotic congenital heart disease or major structural cardiac anomalies were excluded.
② Studies with incomplete data, unavailable full text, or insufficient data for extraction of valid outcome measures;
③ Duplicate publications;
④ Reviews, case reports, conference abstracts, commentaries, and non-randomized controlled studies;
⑤ Animal experiments or *in vitro* cell studies.

### 2.3 Study selection and data extraction

Study selection and data extraction were performed independently by two reviewers. First, titles and abstracts were screened to exclude studies that were clearly ineligible. The full texts of potentially eligible articles were then assessed in accordance with the predefined inclusion and exclusion criteria. The results of study selection were cross-checked by the two reviewers. Any disagreements were resolved through discussion, and, when necessary, adjudicated by a third reviewer.

The following data were extracted: basic study information (first author and year of publication), study design, sample size, baseline characteristics of participants (e.g., sex and gestational age), interventions, and outcome measures.

### 2.4 Quality assessment

The risk of bias of the included randomized controlled trials was assessed using the Cochrane Risk of Bias tool (9). The following domains were evaluated: ① random sequence generation; ② allocation concealment; ③ blinding of participants and personnel; ④ blinding of outcome assessment; ⑤ completeness of outcome data; ⑥ selective outcome reporting; and ⑦ other sources of bias.

Each domain was judged as “low risk”, “high risk”, or “unclear risk”. “Unclear risk” indicated insufficient information or inability to determine the risk of bias. Overall risk of bias was classified as follows: a study was considered to be at high risk of bias if any domain was rated as high risk; at low risk of bias if all domains were rated as low risk; and at unclear risk of bias in all other cases.

### 2.5 Statistical analysis

Statistical analyses were performed using RevMan 5.4. For dichotomous outcomes, effect sizes were expressed as relative risks (RRs) with 95% confidence intervals (CIs). For continuous outcomes, effect sizes were expressed as mean differences (MDs) with 95% CIs. For continuous variables, changes from baseline were preferentially extracted and pooled; when change values were unavailable, post-treatment endpoint values were used for analysis.

Between-study heterogeneity was assessed using Cochran's *Q* test (*χ*² test) and the *I*^2^ statistic. If *I*^2^ was ≤50% and *P* was ≥0.10, heterogeneity was considered low and a fixed-effect model was used for meta-analysis; otherwise, a random-effects model was applied. Where feasible, subgroup analyses were conducted according to treatment regimen, study region, and drug dosage. Sensitivity analyses were performed, where applicable, using a leave-one-out approach to evaluate the robustness of the pooled results. Studies that could not be quantitatively synthesized were summarized descriptively. A two-sided *P* value < 0.05 was considered statistically significant.

## 3 Result

### 3.1 Study selection and characteristics of included studies

**Figure 1:**
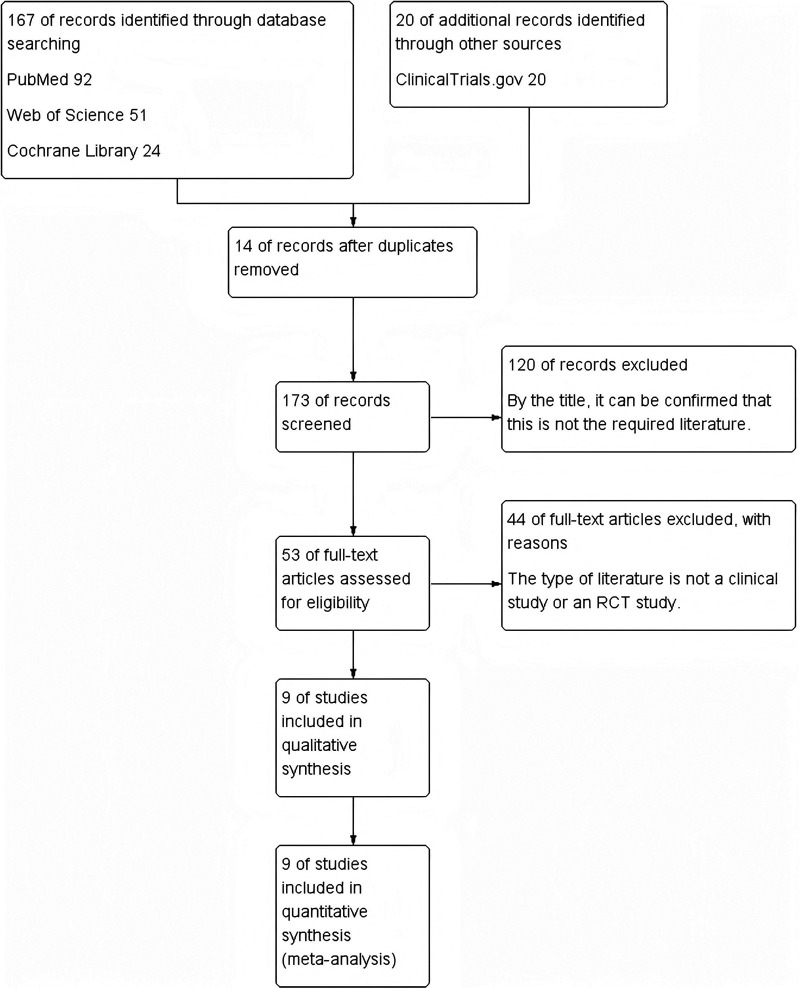
Flow diagram.

A total of 187 records were identified through the systematic search. After screening of titles, abstracts, and full texts, 9 studies (10–18) were included (Figure 1). The studies were published between 2012 and 2025. Most were single-centre studies conducted in Asia; only one was a multicentre randomized placebo-controlled exploratory trial. Study designs were predominantly randomized or controlled clinical studies. Overall, 423 neonates were included, with 215 assigned to bosentan-related treatment groups and 208 to control groups. Interventions included bosentan monotherapy, bosentan plus sildenafil, and bosentan plus inhaled nitric oxide. Control regimens included placebo, sildenafil alone, sildenafil plus beraprost, macitentan plus sildenafil, and other background therapies. Reported outcomes included treatment failure, pulmonary artery pressure, duration of mechanical ventilation, absolute tricuspid regurgitation values and reduction in tricuspid regurgitation at 72 h, and safety outcomes. Study characteristics are shown in Table 1. Oxygenation-related variables, including PaO_2_, oxygen saturation, FiO_2_, and oxygenation index, were reported inconsistently across the included studies. Because of differences in measurement time points, ventilatory support, oxygen supplementation, and reporting formats, these variables were not suitable for quantitative synthesis and were therefore summarized only descriptively where applicable.

**Table 1:** Characteristics of the included studies.

Author, year	Country/region	Study design	Intervention group	Control group	Intervention (n)	Control (n)
Mohamed and Ismail (17)	Saudi Arabia	Randomized, double-blind, placebo-controlled trial	Bosentan	Placebo	24	23
Steinhorn et al. 2016 (18)	Multicenter (USA/Europe/Australia/Asia)	Randomized, multicenter, placebo-controlled exploratory trial	Bosentan + inhaled nitric oxide (iNO)	Placebo + inhaled nitric oxide (iNO)	13	8
Fatima et al. (11)	Pakistan	Single-blind clinical trial	Sildenafil + bosentan	Sildenafil alone	50	50
Maneenil et al. (16)	Thailand	Single-blind clinical trial	Bosentan + inhaled nitric oxide (iNO)	Placebo + inhaled nitric oxide (iNO)	15	14
Farhangdoust et al. (12)	Iran	Double-blind clinical trial	Bosentan	Sildenafil	15	25
Adnan et al. (10)	Pakistan	Open-label, non-randomized quasi-experimental study	Sildenafil + bosentan	Sildenafil + beraprost	25	25
Kumar et al. (5)	India	Controlled study (described by authors as randomized)	Sildenafil + bosentan	Sildenafil	25	15
Kallimath et al. (13)	India	Single-center, open-label randomized controlled trial	Oral bosentan	Oral sildenafil	18	18
Kashaki et al. (14)	Iran	Randomized, double-blind, non-inferiority parallel trial	Bosentan + sildenafil	Macitentan + sildenafil	30	30

### 3.2 Analysis of therapeutic efficacy

Oxygenation-related variables, including PaO₂, oxygen saturation, FiO₂ requirement, oxygenation index, duration of oxygen therapy, and the ability to wean iNO, were reviewed across the included studies. However, these outcomes were reported inconsistently, with differences in measurement time points, baseline oxygen requirement, ventilatory support, use of iNO, and reporting formats. Therefore, a quantitative comparison of oxygen therapy requirements or iNO weaning between bosentan-related therapy and non-bosentan groups was not feasible.

#### 3.2.1 Treatment failure

**Figure 2:**
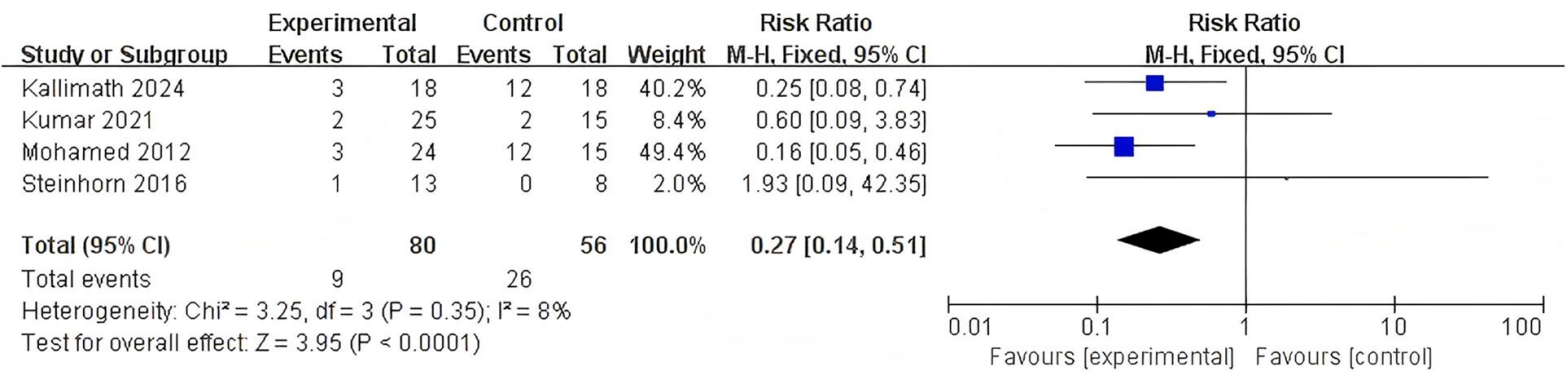
Forest plot of the treatment failure rate.

A total of 4 studies (5, 13, 17, 18) reported treatment outcomes. Between-study heterogeneity was low (*I*^2^ = 8%, *P* = 0.35); therefore, a fixed-effect model was used for meta-analysis. The pooled analysis showed that bosentan-related therapy was associated with a significantly lower treatment failure rate than control treatment (RR = 0.27, 95% CI: 0.14–0.51, *P* < 0.0001). Across the included studies, treatment failure occurred in 9 of 80 neonates in the bosentan group and in 26 of 56 neonates in the control group. These findings suggest that bosentan may significantly reduce the risk of treatment failure in neonates with PPHN (Figure 2).

#### 3.2.2 Change in pulmonary artery pressure

**Figure 3:**
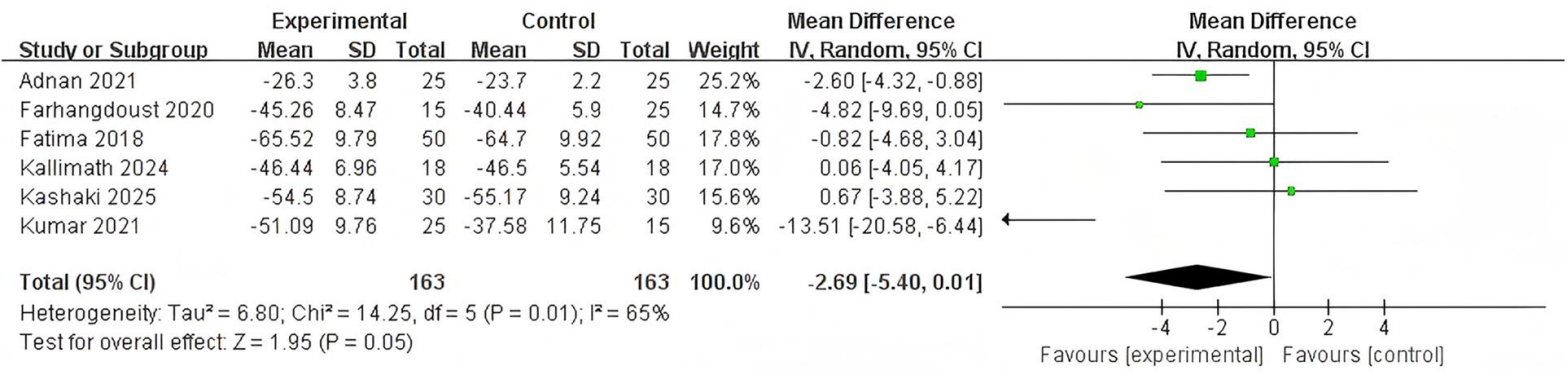
Forest plot of the change in pulmonary artery pressure.

A total of 6 studies (5, 10–14) reported pulmonary pressure-related outcomes. These outcomes were extracted as reported by the original studies and were based on non-invasive echocardiographic assessment rather than invasive catheterization. Across studies, pulmonary pressure was reported using related but not fully identical measures, including pulmonary artery pressure, pulmonary artery systolic pressure, or tricuspid regurgitation-derived estimates of pulmonary pressure. When the original study reported tricuspid regurgitation-derived values, these were treated as echocardiography-derived pulmonary pressure estimates. For studies that reported pulmonary artery pressure or pulmonary artery systolic pressure without sufficient methodological details, the values were retained as author-reported pulmonary pressure estimates and interpreted cautiously. Heterogeneity was present across studies (*I*^2^ = 65%, *P* = 0.01); therefore, a random-effects model was used for meta-analysis. The pooled analysis showed that the reduction in pulmonary artery pressure was greater in the bosentan-related treatment group than in the control group, with borderline statistical significance (MD = −2.69, 95% CI: −5.40 to 0.01, *P* = 0.05). These findings suggest that bosentan-related therapy may be associated with more favorable changes in pulmonary pressure-related hemodynamic estimates in neonates with PPHN, although the results should be interpreted with caution because pulmonary arterial pressure is an indirect and flow-dependent parameter rather than a direct measure of pulmonary vascular resistance (Figure 3).

#### 3.2.3 Length of hospital stay

**Figure 4:**
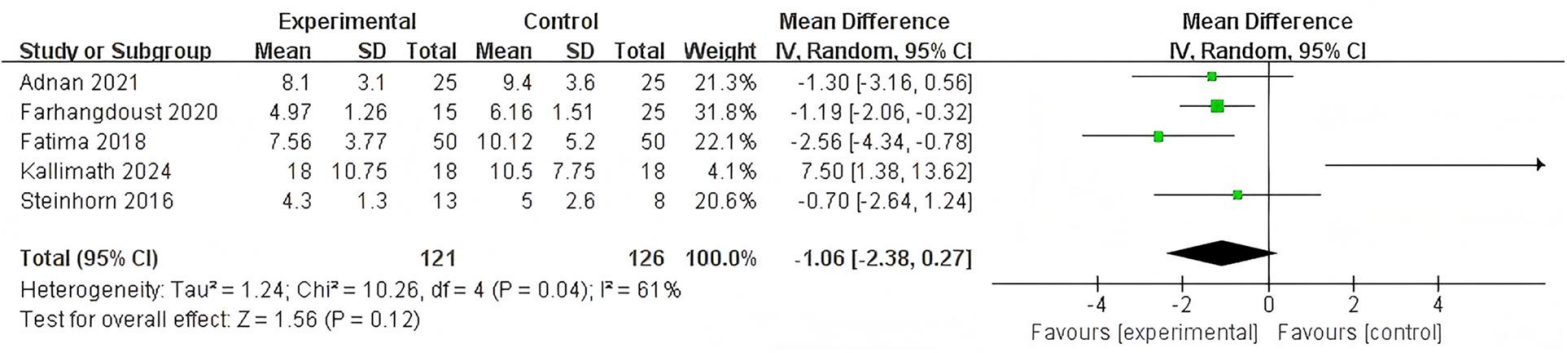
Forest plot of length of hospital stay in neonates with PPHN.

A total of 5 studies (10–13, 18) reported length of hospital stay. Moderate heterogeneity was observed across studies (*I*^2^ = 61%, *P* = 0.04). Therefore, a random-effects model was used for meta-analysis. The pooled analysis showed a trend toward a shorter length of hospital stay in the bosentan-related treatment group than in the control group, but the difference was not statistically significant (MD = −1.06, 95% CI: −2.38 to 0.27, *P* = 0.12). These findings suggest that the current evidence is insufficient to demonstrate a significant reduction in hospital stay with bosentan-related therapy in neonates with PPHN (Figure 4).

#### 3.2.4 Duration of mechanical ventilation

**Figure 5:**
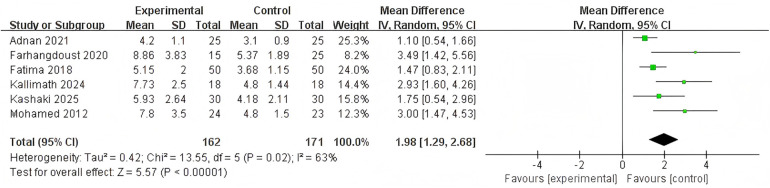
Forest plot of duration of mechanical ventilation.

A total of 6 studies (10–14, 17) reported the duration of mechanical ventilation. Heterogeneity was present across studies (*I*^2^ = 63%, *P* = 0.02); therefore, a random-effects model was used for meta-analysis. The pooled analysis showed that the duration of mechanical ventilation was significantly longer in the bosentan-related treatment group than in the control group (MD = 1.98, 95% CI: 1.29 to 2.68, *P* < 0.00001). These findings do not support a benefit of bosentan-related therapy in shortening the duration of mechanical ventilation in neonates with PPHN and may suggest an association with a longer duration of mechanical ventilation (Figure 5).

#### 3.2.5 Absolute tricuspid regurgitation values at 72 h

**Figure 6:**
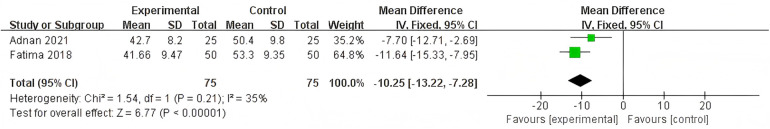
Forest plot of absolute tricuspid regurgitation values at 72 h.

A total of 2 studies (10, 11) reported absolute tricuspid regurgitation values at 72 h. Between-study heterogeneity was low (*I*^2^ = 35%, *P* = 0.21), and a fixed-effect model was therefore used for meta-analysis. The pooled analysis showed that absolute tricuspid regurgitation values at 72 h were lower in the bosentan-related treatment group than in the control group (MD = −10.25, 95% CI: −13.22 to −7.28, *P* < 0.00001), suggesting that bosentan-related therapy may be associated with lower tricuspid regurgitation-derived pulmonary pressure estimates at 72 h (Figure 6).

#### 3.2.6 Reduction in tricuspid regurgitation at 72 h

**Figure 7:**
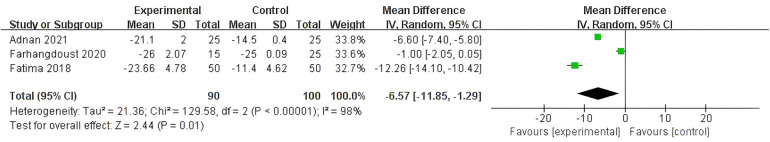
Forest plot of the reduction in tricuspid regurgitation at 72 h.

A total of 3 studies (10–12) reported the reduction in tricuspid regurgitation at 72 h, and all used bosentan at 1 mg/kg per dose twice daily (BID). Heterogeneity was high (*I*^2^ = 98%, *P* < 0.00001), and a random-effects model was therefore applied. The pooled analysis showed a greater reduction in tricuspid regurgitation at 72 h in the bosentan-related treatment group than in the control group (MD = −6.57, 95% CI: −11.85 to −1.29, *P* = 0.01), suggesting a potential advantage of bosentan-related therapy in improving echocardiographic hemodynamic parameters in neonates with PPHN (Figure 7).

### 3.3 Adverse events

Adverse events were reported in 7 studies (5, 11–14, 17, 18). Because the types of adverse events varied across studies, quantitative synthesis was not feasible; therefore, a descriptive analysis was performed. *Steinhorn* et al. (18) reported 3 cases of generalized edema, 3 cases of anemia, 2 cases of vomiting, and 2 cases of hepatitis (abnormal liver function) in the bosentan group, whereas the control group had 2 cases of pneumothorax, 1 case of sepsis, and 1 case of anemia. *Kallimath* et al. (13) reported 2 cases of systemic hypotension in the bosentan group; in the control group, only 1 case of systemic hypotension was observed, along with 1 case of shock secondary to multisystem inflammatory syndrome, and 1 death was reported. *Fatima* et al. (11), *Farhangdoust* et al. (12), *Mohamed* et al. (17), and *Kashaki* et al. (14) all reported no apparent adverse events such as hypotension, gastrointestinal intolerance, or hepatic or renal dysfunction after bosentan treatment. *Kumar* et al. (5) found 2 cases of abnormal liver function in the bosentan-based combination therapy group, whereas no abnormal liver function or similar adverse events were reported in the sildenafil monotherapy group; 2 deaths occurred in each group.

### 3.4 Bias analysis

**Figure 8:**
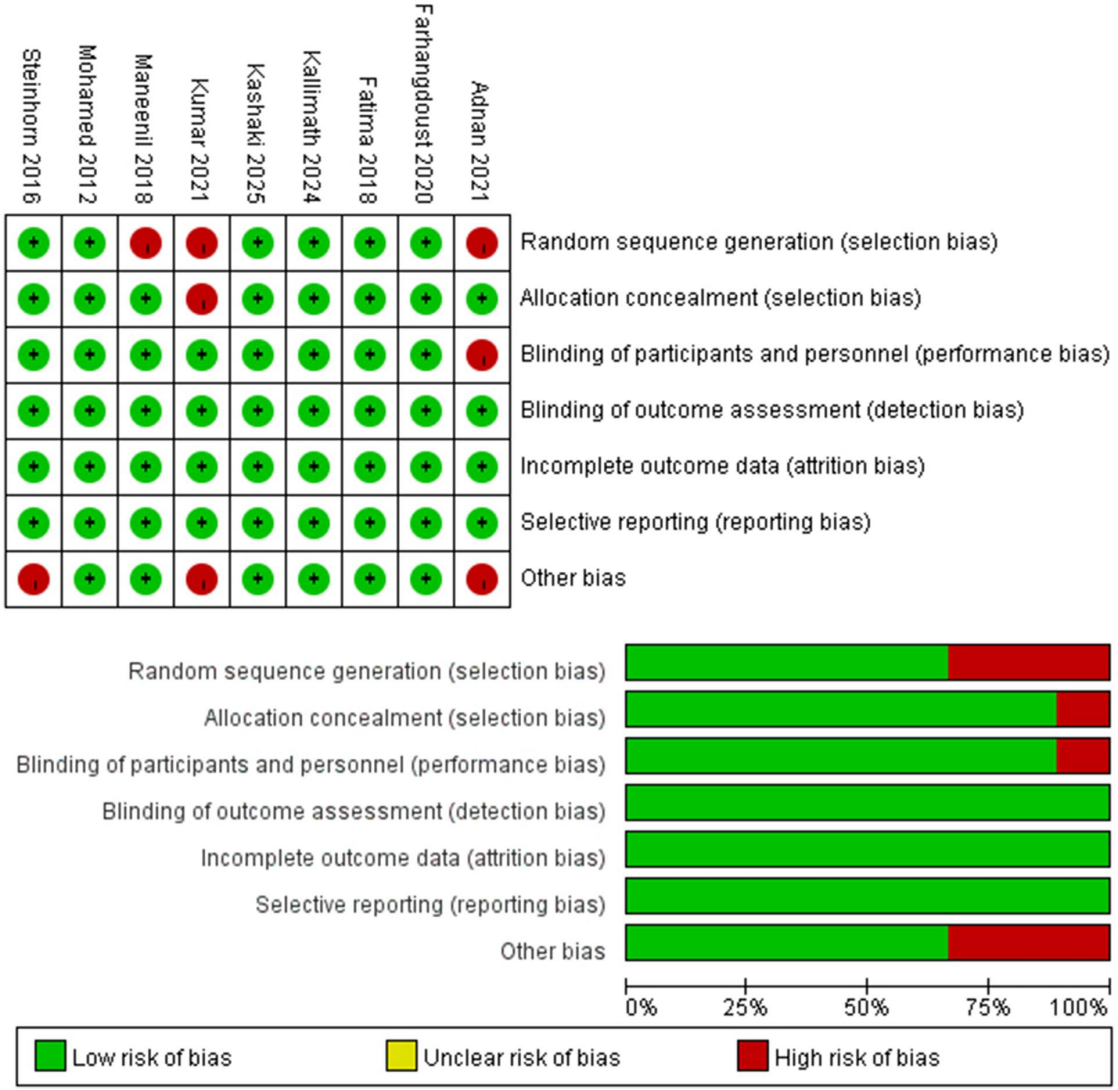
Risk-of-bias summary of the included studies.

The methodological quality of the included studies was assessed using the Cochrane risk-of-bias tool. Overall, the methodological quality of the included studies was acceptable. Most studies were judged to be at low risk of bias for blinding of outcome assessment, incomplete outcome data, and selective reporting, whereas random sequence generation, allocation concealment, blinding of participants and personnel, and other sources of bias were the main potential sources of bias. Some studies were judged to be at high risk of bias because of non-random group allocation, open-label design, or other methodological limitations (Figure 8).

**Figure 9:**
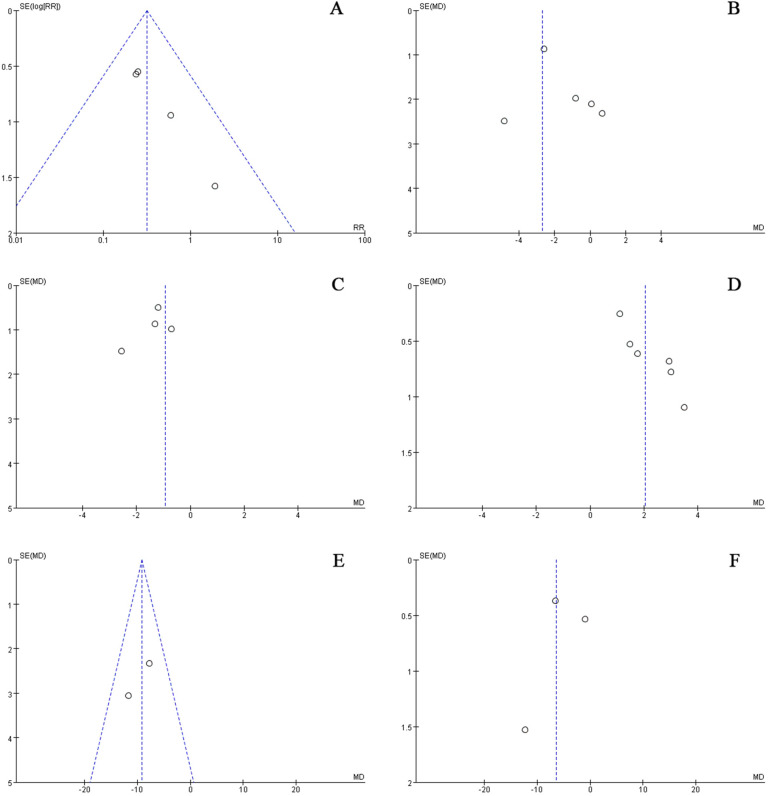
Funnel plots for publication bias across outcomes. (**A**-**F**) Funnel plots for treatment failure, pulmonary artery pressure, duration of mechanical ventilation, length of hospital stay, absolute tricuspid regurgitation at 72h, and reduction in tricuspid regurgitation at 72h, respectively.

Funnel plots were used for exploratory visual assessment of potential publication bias for treatment failure, change in pulmonary artery pressure, length of hospital stay, duration of mechanical ventilation, absolute tricuspid regurgitation values at 72 h, and reduction in tricuspid regurgitation at 72 h (Figures 9A–F). In a funnel plot, each point represents one included study. A relatively symmetrical distribution of studies around the pooled effect estimate suggests less visual concern for publication bias or small-study effects, whereas an uneven or asymmetrical distribution may suggest possible publication bias, small-study effects, or clinical and methodological heterogeneity. In the present analysis, the funnel plots for treatment failure, change in pulmonary artery pressure, and duration of mechanical ventilation showed some asymmetry, indicating that these outcomes may have been influenced by small-study effects, selective reporting, or between-study heterogeneity. The funnel plot for length of hospital stay was relatively more concentrated, but the small number of studies still limited interpretation. The funnel plots for absolute tricuspid regurgitation values and reduction in tricuspid regurgitation at 72 h were particularly difficult to interpret because only a very small number of studies contributed to these outcomes. Therefore, all funnel plot findings in this meta-analysis should be interpreted only as exploratory visual assessments rather than formal evidence of publication bias.

## 4 Discussion

PPHN is characterized pathologically by persistently elevated pulmonary vascular resistance, increased pulmonary vascular reactivity, abnormal pulmonary vascular smooth muscle remodeling, and reduced angiogenesis (19). Current management of PPHN is based on supportive care and comprehensive strategies including lung recruitment, optimization of ventilation, and the use of pulmonary vasodilators, with the primary goals of reducing pulmonary vascular resistance, improving oxygenation, and facilitating the normal transition from fetal to postnatal circulation (20).

Endothelin (ET) is a potent vasoconstrictive peptide produced by vascular endothelial cells. Increased ET-A-mediated vasoconstriction and reduced ET-B-mediated vasodilation are thought to contribute to the development and progression of PPHN (21). As a dual endothelin receptor antagonist, bosentan may attenuate endothelin-mediated pulmonary vasoconstriction and thereby reduce pulmonary vascular tone or resistance. However, pulmonary arterial pressure and pulmonary vascular resistance index are not interchangeable parameters. Pulmonary arterial pressure may be influenced not only by pulmonary vascular resistance, but also by pulmonary blood flow, shunt direction and magnitude, left atrial pressure, systemic hemodynamics, and the severity of parenchymal lung disease. For example, increased shunt flow may elevate pulmonary arterial pressure even when pulmonary vascular resistance is not markedly increased, whereas severe lung disease may increase pulmonary vascular resistance without a proportional increase in measured pulmonary arterial pressure. Therefore, the pulmonary pressure-related outcomes in this meta-analysis should be interpreted as echocardiography-derived estimates of pulmonary hemodynamic change, rather than direct evidence of reduced pulmonary vascular resistance index.

The present meta-analysis showed that bosentan-related therapy significantly reduced the treatment failure rate in neonates with PPHN (RR = 0.27, 95% CI: 0.14–0.51, *P* < 0.0001). For hemodynamic outcomes, absolute tricuspid regurgitation values at 72 h were lower in the bosentan-related treatment group than in the control group (MD = −9.15, 95% CI: −12.79–−5.52, *P* < 0.00001), and the reduction in tricuspid regurgitation at 72 h was greater in the bosentan-related treatment group (MD = −6.42, 95% CI: −11.30–−1.54, *P* = 0.01). The reduction in pulmonary artery pressure also favored the bosentan-related treatment group overall, although the difference was only of borderline statistical significance (MD = −2.69, 95% CI: −5.40–0.01, *P* = 0.05). These findings suggest that bosentan-related therapy may be associated with favorable changes in echocardiography-derived pulmonary pressure estimates and a lower short-term risk of treatment failure. This is consistent with the findings of *Goissen* et al. (22) and *Nakwan* et al. (23). With regard to preterm infants, only case reports are currently available, suggesting that pulmonary artery pressure and oxygen saturation may improve after bosentan treatment in extremely preterm infants with PPHN. *Radicioni* et al. (24) reported improvement in pulmonary artery pressure and oxygen saturation in a 28-week preterm infant with PPHN after bosentan treatment. Therefore, we can only conclude that the potential benefit of bosentan across different gestational ages warrants further exploration, but the available evidence is insufficient to infer that its efficacy is unaffected by gestational age. One included trial directly compared macitentan plus sildenafil with bosentan plus sildenafil in clinically stable neonates with PPHN. However, macitentan-related evidence was limited to this single study, with only 30 neonates in each treatment group, and both groups received concomitant sildenafil. Therefore, the available data were insufficient to support a reliable subgroup analysis or definitive conclusions regarding the comparative efficacy or safety of macitentan vs. bosentan in neonatal PPHN. These findings should be interpreted as exploratory and hypothesis-generating.

Notably, for clinical course outcomes, bosentan-related therapy did not significantly shorten length of hospital stay (MD = −1.06, 95% CI: −2.38–0.27, *P* = 0.12), and the duration of mechanical ventilation was longer than in the control group (MD = 2.04, 95% CI: 1.26–2.82, *P* < 0.00001). These findings should not be interpreted solely as direct markers of pulmonary vasodilator efficacy. Length of hospital stay is determined by multiple factors beyond pulmonary pressure alone, including the underlying cause of PPHN, recovery from parenchymal lung disease, duration of respiratory and oxygen support, feeding tolerance, resolution of infection or other comorbid conditions, adequate growth, and local discharge criteria. Similarly, the duration of mechanical ventilation is a multifactorial clinical outcome influenced not only by changes in pulmonary vascular tone, but also by the underlying etiology and phenotype of PPHN, the severity of respiratory failure, the extent of parenchymal lung disease, ventilatory strategy, sedation practice, weaning protocols, and center-specific extubation criteria. Therefore, improvement in pulmonary pressure-related estimates may not necessarily translate into shorter hospitalization or earlier extubation, particularly when clinical recovery is limited by lung parenchymal disease or non-standardized respiratory management. In addition, PaO₂ alone may be difficult to interpret as a treatment-response outcome in neonates with PPHN because it is influenced by the level of respiratory support, FiO₂, shunt direction and magnitude, and the severity of parenchymal lung disease. Oxygenation outcomes may therefore be better interpreted using more integrated measures, such as oxygenation index, FiO₂ requirement, duration of oxygen therapy, or the ability to wean iNO, when consistently reported. Previous studies have shown (25, 26) that the duration of mechanical ventilation may differ substantially across different etiologic subtypes of PPHN. Thus, improvement in pulmonary pressure-related estimates may not necessarily translate into earlier extubation, particularly when respiratory recovery is limited by lung parenchymal disease or non-standardized respiratory management. *Tsoi et al*. (27) classified PPHN into perinatal etiologies and fetal developmental etiologies, and found that the disease course resolved more rapidly in the perinatal etiology group; by day 10 after birth, the proportion of infants who had been extubated was significantly higher in that group (75% vs. 37%, *P* < 0.01), indicating that different etiologies/phenotypes of PPHN are indeed associated with marked differences in ventilation-related clinical course outcomes. Second, in a multicenter randomized placebo-controlled trial, *Steinhorn* et al. (18) noted that bosentan did not improve oxygenation or mechanical ventilation-related outcomes, which might have been related to delayed enteral absorption of bosentan during the early treatment phase in critically ill neonates or to greater illness severity among infants receiving bosentan. *Kallimath* et al. (13) further showed that, compared with sildenafil, bosentan had a slower onset of effect in reducing PASP and FiO₂ and was more frequently associated with the need for additional pulmonary vasodilators. Therefore, the finding of a longer duration of mechanical ventilation should be interpreted with caution and is insufficient to directly suggest a detrimental effect of bosentan on this outcome.

Mechanistically, bosentan binds to both ET-A and ET-B receptors, thereby reducing receptor-mediated vasoconstrictive activity and enhancing vasodilatory effects, ultimately promoting pulmonary vasodilation, lowering pulmonary artery pressure, and improving oxygenation (28). However, the overall sample size of the studies included in this meta-analysis was small, most studies were single-center investigations from Asia, and the majority of enrolled infants were term or near-term neonates; therefore, the generalizability of the current evidence remains limited.

With regard to safety, hepatic toxicity deserves specific consideration because elevated aminotransferase levels are a recognized safety concern during bosentan therapy and are routinely considered in clinical counselling and monitoring. In the present meta-analysis, hepatic adverse events were reported inconsistently across studies. Only a small number of liver-related events were identified, including hepatitis or abnormal liver function in the bosentan group in *Steinhorn* et al. (18). and abnormal liver function in the bosentan-based combination therapy group in *Kumar* et al. (5) Several other studies reported no apparent hepatic or renal dysfunction after bosentan treatment. However, the absence of reported hepatic events should not be interpreted as evidence of no risk, because most included studies had small sample sizes, short follow-up periods, and non-standardized liver enzyme monitoring or reporting. In addition, critically ill neonates with PPHN may have multiple alternative contributors to liver enzyme abnormalities, including hypoxemia, systemic hypoperfusion, sepsis, parenteral nutrition, and concomitant medications. Therefore, the current evidence suggests no clear signal of frequent severe hepatic toxicity, but it remains insufficient to determine whether bosentan-related therapy has a low hepatic risk or a hepatic risk comparable to control therapy. Future trials should prospectively report alanine aminotransferase, aspartate aminotransferase, bilirubin, timing of liver function testing, severity grading, treatment interruption, and resolution of hepatic abnormalities. Previous studies have suggested that bosentan-related hepatotoxicity may be associated with inhibition of bile salt transport and dose-/exposure-related mechanisms; in addition, edema and anemia are also recognized safety signals in adults and children receiving bosentan for pulmonary arterial hypertension. In the present meta-analysis, 7 studies reported adverse events after treatment, but quantitative synthesis was not performed because of substantial differences in the types of adverse events reported. Regarding the safety of bosentan in neonates, case reports and case series have generally suggested acceptable short-term tolerability, although studies specifically focusing on drug-related adverse events remain scarce. *Nakwan* et al. (23) reported that a neonate with severe PPHN showed marked improvement in oxygenation after adjunctive bosentan therapy, without any clearly identified serious drug-related adverse events. *Radicioni* et al. (24) reported improvement in pulmonary hypertension in a 28-week preterm infant after bosentan was added to inhaled nitric oxide and sildenafil, again without clearly documented severe adverse effects. *Pawar* et al. (29) reported that a neonate with postoperative pulmonary hypertension improved after the addition of bosentan, and no obvious drug-related adverse effects were observed during treatment. Overall, the currently available evidence suggests that bosentan is generally well tolerated in the short term in neonates; however, clinicians should remain vigilant for hypotension, anemia, edema, and abnormal liver function, and should closely monitor both hepatic function and hemodynamic status during treatment.

This meta-analysis has several limitations.

(1). The overall sample size of the included studies was small, and only a limited number of studies were available for several outcomes, which may have affected the stability of the pooled estimates.
(2). The types and reporting formats of adverse events varied substantially, precluding quantitative synthesis and limiting the strength of the safety evidence.
(3). There was substantial heterogeneity across the included studies in terms of intervention regimens, control measures, drug dosages, outcome definitions, and underlying PPHN etiologies. In particular, etiologically distinct phenotypes such as meconium aspiration syndrome-related PPHN and congenital diaphragmatic hernia-related PPHN could not be analyzed separately because etiology-specific outcome data were not consistently available.
(4). Most studies were single-center investigations conducted in Asia, and some had methodological limitations such as non-random group allocation, open-label design, or other sources of bias, which may have affected both the generalizability and the strength of the conclusions.

In addition, only one included study evaluated macitentan as a comparator, with a small number of macitentan-exposed neonates. Therefore, no reliable subgroup analysis or comparative conclusion regarding macitentan could be made.

## 5 Conclusion

Taken together, the available evidence suggests that bosentan-related therapy may be associated with a lower risk of treatment failure and favorable changes in echocardiography-derived pulmonary pressure estimates in neonates with PPHN. However, these pulmonary pressure-related findings should be interpreted as indirect hemodynamic estimates rather than direct evidence of reduced pulmonary vascular resistance. Evidence for benefit in more clinically definitive outcomes, such as length of hospital stay, remains limited, and no advantage has been shown in shortening the duration of mechanical ventilation. Bosentan may represent a potential adjunctive option for selected neonates with PPHN, particularly in settings where inhaled nitric oxide (iNO) is unavailable or insufficiently effective. Nevertheless, its efficacy and safety require further evaluation in larger, well-designed randomized controlled trials with standardized diagnostic criteria, etiologic stratification, and consistent reporting of oxygenation, echocardiographic, and safety outcomes, particularly hepatic adverse events and liver enzyme monitoring.

## Data Availability

The datasets presented in this study can be found in online repositories. The names of the repository/repositories and accession number(s) can be found in the article/Supplementary Material.
