# Supplementary material for: Efficacy and safety of bosentan-related therapy in neonates with persistent pulmonary hypertension of the newborn: a systematic review and meta-analysis
Source: Front Pediatr. 2026 Jun 3;14:1836276. doi: 10.3389/fped.2026.1836276 (PMC13272431; doi:10.3389/fped.2026.1836276)
Supplement: Supplementary file 1 [file Table1.docx]

Supplementary Table S1 Search Strategy

| database | Search Strategy |
| --- | --- |
| Cochrane library | #1 "persistent pulmonary hypertension of the newborn" OR PPHN OR "persistent fetal circulation" OR "persistent pulmonary hypertension" OR "pulmonary hypertension"  #2 bosentan OR Tracleer  #3 newborn OR newborns OR neonate OR neonates OR neonatal OR infant OR infants  #4 #1 AND #2 AND #3 |
| PubMed | (("Hypertension, Pulmonary"[Mesh] OR "persistent pulmonary hypertension of the newborn"[tiab] OR PPHN[tiab] OR "persistent fetal circulation"[tiab]  OR "persistent pulmonary hypertension"[tiab] OR "pulmonary hypertension"[tiab]))AND(("Bosentan"[Mesh] OR bosentan[tiab]  OR Tracleer[tiab] OR "Ro47-0203"[tiab]))  AND(newborn[tiab] OR newborns[tiab] OR neonate[tiab] OR neonates[tiab]  OR neonatal[tiab] OR infant[tiab] OR infants[tiab]OR "Infant, Newborn"[Mesh]) |
| Web of Science | TS=("persistent pulmonary hypertension of the newborn"  OR PPHN  OR "persistent fetal circulation"  OR "persistent pulmonary hypertension")  AND TS=(bosentan OR Tracleer OR "Ro47-0203")  AND TS=(newborn OR newborns OR neonate OR neonates OR neonatal OR infant OR infants) |
